# Health extension workers contribution on tuberculosis case notification in Tigray region, Northern Ethiopia: A concurrent mixed method study

**DOI:** 10.1371/journal.pone.0271968

**Published:** 2022-08-16

**Authors:** Hailay Gebretnsae, Tsegay Hadgu, Brhane Gebrekidan Ayele, Alemnesh Abraha, Equbay Gebre-egziabher, Mulugeta Woldu, Tsegay Wellay, Gebregziabher Berihu Gebrekidan, Measho Gebreslassie Gebregziabher

**Affiliations:** 1 Tigray Health Research Institute, Mekelle, Tigray, Ethiopia; 2 College of Health Sciences, Mekelle University, Mekelle, Ethiopia; Oregon State University, UNITED STATES

## Abstract

**Background:**

Despite the emphasis placed on Community Based Tuberculosis Care (CBTC) implementation by Health Extension Workers (HEWs) within the National Tuberculosis Program (NTP) in Ethiopia, there is little evidence on contribution of HEWs on TB case notification. Therefore, this study aimed to describe the contribution of HEWs on TB case notification and its associated factors in Tigray region, Northern Ethiopia.

**Methods:**

A concurrent mixed method (quantitative and qualitative) cross-sectional study design was conducted in three randomly selected districts in Tigray region, Northern Ethiopia. Quantitative data were collected using a pre-tested semi-structured questionnaire. Qualitative data were collected using Focused Group Discussions (FGDs) and Key Informant Interviews (KIIs) to further describe the community participation and presumptive TB identification and referral system. For the quantitative data, binary logistic regression analysis was done and all variables with P-value of < 0.25 in bivariate analysis were included in the multi-variable model to see predictors of HEWs contribution to TB notification. The qualitative data were thematically analyzed using Atlas.ti version 7.

**Results:**

In this study, a total of 68 HEWs were included. From March 1, 2017 to February 28, 2018, a total of 427 TB cases notified in the study areas and one-third (34%) of them were notified by the HEWs referral. Provision of Community Based-Directly Observed Treatment Short course (CB–DOTS) (Adjusted Odds Ratio (AOR) = 3.63, 95% Confidence Interval (CI) = 1.18–11.19) and involvement of community volunteers on CBTC (AOR = 3.31, 95% CI = 1.10–10.09) were significantly associated with the contribution of HEWs on TB case notification. The qualitative findings indicated that high workload of HEWs, inaccessibility of TB diagnostic services at nearby health facilities, and transportation and investigation costs were identified as factors affecting for presumptive TB referral by HEWs.

**Conclusions:**

Provision of CB-DOTS and involvement of community volunteers in CBTC activities should be strengthened to improve the HEWs contribution on TB case notification. Additionally, HEWs should be empowered and further interventions of TB diagnostic services at diagnostic health facilities are needed to improve presumptive TB referral by HEWs.

## Introduction

In 2017, an estimated 10 million people around the world became ill with Tuberculosis (TB), and 1.3 million died from it. Ethiopia is still among the 30 high TB, TB/HIV and MDR-TB burden countries in the world [1]. Global END TB Strategy is adopted and engagement of communities is one of the core components [2]. Globally an average of 27% notified TB cases attributed to community referrals in 2017 [1].

In the majority of TB endemic countries in the world, Passive Case Finding (PCF) has been used to identify TB cases. However, this strategy is inadequate to detect the large burden of undiagnosed TB in the community. It is estimated that in 2017, more than 3.6 million people who developed TB were ‘‘missed” by the health system in the world [1]. Active Case Finding (ACF) for TB has been recognized as an important complementary strategy to PCF in order to early diagnose and treat TB patients [3].

Engagement of communities includes a wide range of activities that contribute to TB prevention, diagnosis, treatment and care that are carried out by Community Health Workers (CHWs) and Community Volunteers (CVs) [4]. A CHW is a person with formal education who is trained to contribute to community-based health services, as well as TB prevention and control. A CV is a community member who has been systematically sensitized about health service including TB prevention and control, either through a short specific training scheme or through repeated contact with health professionals [5,6]. Evidence suggests that using CHWs in health programmes could be a cost-effective intervention, particularly for TB programme [7]. In some countries for example Ethiopia, CHWs are an integral part of the health system and privileges of formal employment [4].

In 2003, Ethiopia launched a Health Extension Programme (HEP) to address primary health service coverage to rural communities. Since 2004, Health Extension Workers (HEWs) are CHWs who have been trained by the federal ministry of health to implement the HEP in kebele (the lowest administrative unit with an average population of 5000) and serve at a health post [8,9]. In 2011, a community volunteers called Women’s Development Army (WDA) strategy was introduced. WDA team is the smallest community organization unit and consists of 30 women residing in a neighborhood in a one-to five networks (with one leader and five members) [10].

In Ethiopia, Community Based TB Care (CBTC) is among the sixteen health extension packages being implemented at health post and community level. It includes community awareness creation, presumptive TB case identification and referral, Community Based—Directly Observed Treatment Short course (CB–DOTS) and follow up [11]. HEWs identify individuals with presumptive TB at health post and during their home to home visits and refer to the nearest diagnostic health facility for further investigation [12]. HEWs are linked to the community through a network of community volunteers (WDA). Involvement of WDA team is fostering early identification and referral of presumptive TB cases in the community [13].

In Ethiopia, the presence of HEWs in the community could increase access to TB diagnostic and treatment services [14,15], and ACF intervention through HEWs in the community could improve the TB case detection rate [16–18]. However, the routine household contacts screening and TB cases notification by HEWs remains low [19,20].

Despite the emphasis placed on CBTC implementation by HEWs within the National TB program (NTP) in Ethiopia [13], there is little evidence on contribution of HEWs on TB notification [20]. Sharing the experiences of HEWs contribution on TB case notification will contribute to inform policy direction to achieve End TB strategy targets. Therefore, this study aimed to describe the contribution of HEWs on TB case notification and its associated factors in Tigray region, Northern Ethiopia.

## Methods

### Study design and setting

A concurrent mixed method (quantitative and qualitative) cross-sectional study design was carried out from March 12, 2018 to April 7, 2018 in three selected districts of Tigray region, Northern Ethiopia. Tigray region is one of the nine regional states of Ethiopia located in the north. The region has seven zones which are further divided into 52 districts (34 rural and 18 urban) and 799 Kebeles (722 rural and 77 urban). In 2018, there were 712 health posts, 216 health centers and 39 hospitals that provide health services to the community.

### Sampling procedures and participant recruitment

We randomly selected three districts using lottery method from 52 districts in Tigray region, Northern Ethiopia. All (fifty–four) health posts with at least one TB case from March 1, 2017 to February 28, 2018 in the three randomly selected districts were included in the study. In each selected health post, all (sixty–eight) HEWs who had their own separate CBTC activities report and were working from March 1, 2017 to February 28, 2018 at the health posts were recruited in the study. In case of we found more than one eligible HEWs, we included all of them in the study. Health posts that had not at least one TB case and/or eligible HEW during specified period were excluded from this study (S1 Fig).

Furthermore, three Focused Group Discussions (FGDs) (with 6–8 members each) with WDA leaders and seven Key Informant Interviews (KIIs) with HEWs and Primary Health Care Unit (PHCU) TB focal persons, supervisors and directors were conducted to describe the community participation, and presumptive TB identification and referral system. The FGDs and KIIs participants were purposively selected based on their potential wealth of information on CBTC implementation and their sample sizes were determined by data saturation level.

### Data collection technique and data quality assurance

A pre-tested semi-structured questionnaire was used to collect quantitative data (S1 File). Data were collected using a face-to-face interview with eligible HEWs about socio-demographic, knowledge of HEWs on TB, implementation of CBTC activities, availability and utilization of TB related reporting and recording tools. In addition, data on the number of presumptive TB cases referred by HEWs and the number of cases diagnosed with TB were reviewed from the health posts’ registers from March 1, 2017 to February 28, 2018. Observational technique also was used to check the availability and utilization of reporting and recording tools. Ten trained diploma nurses who had an experience on data collection were recruited to collect the quantitative data. Furthermore, a pre-tested FGD and KII interview guides were used to generate qualitative data (S2 and S3 Files). Face-to-face interviews were conducted in a convenient and confidential area using tape recorder and field note by three principal investigators who had an experience and training on qualitative data collection. Transcription and preliminary analysis were done during data collection period. Debriefing sessions were conducted to have a common understanding and to include new probing questions in the interview guide. Completeness and checking for data quality was done by supervisors and principal investigators on a daily basis.

### Measurement of variables

#### Proportion of HEWs contribution on TB case notification

This refers to percentage of TB cases (all forms) referred by the HEWs among all TB cases notified from March 1, 2017 to February 28, 2018. The numerator was number of registered TB cases (all forms) initially referred by the HEWs to a health facility for diagnosis during the specified reporting period and the denominator was total number of all forms of TB cases registered during same reporting period [11].

#### HEWs contribution on TB case notification

This dichotomous dependent variable was labeled as value of 1 = Yes, if the HEW notified at least one TB case (all forms) and 0 = No, if the HEW failed to notify any TB case during the specified reporting period.

#### Knowledge of HEWs on TB

This variable was computed from five knowledge related questions (cause, mode of transmission, cardinal symptoms, diagnostic methods and targeted groups for systematic screening of TB). Those scored greater than the mean were considered as have good knowledge and those who scored below the mean were considered as have poor knowledge.

#### Involvement of community volunteers on CBTC activities

This dichotomous variable was labeled as value of 1 = Yes, if WDA/Kebele leader actively involved either in one or more of the following CBTC activities: conducted social mobilization on TB prevention and control, provided health education, identified and promoted presumptive TB referral, and supported TB treatment and participated in other TB related activities (provided financial support for TB cases and reviewed TB activities performance).

### Data management and analysis

Quantitative data were entered using Epi-Info version 3.5.1 software and then exported to SPSS version 21 for analysis. Descriptive statistics was carried out using frequency tables and graphs to describe the characteristics of the participants. Binary logistic regression analysis was done to identify predictor variables for the contribution of HEWs on TB case notification. All variables with p-value < 0.25 in the bivariate analysis were included in multi-variables logistic regression model and variables which had a p-value less than 0.05 were considered statistically significant in the final model.

Qualitative data collection and analysis were conducted concurrently. The first three authors (HG, TH and BGA) transcribed and translated the audio taped data. Each audio taped data were listened repeatedly and transcribed verbatim. HG carried out the data coding and categorization using Atlas. ti software version 7. Field notes and investigator’s memo were also linked to the respective files in the software during analysis. Codes were developed from the respondents’ ideas and then it was synthesized in to families. Finally, themes were developed from the families. All authors reviewed the list of codes, families and themes, and further refinement was made through discussion.

### Ethical consideration

The ethical clearance of the study was obtained from the institutional review committee of Tigray Health Research Institute (Reference number: THRI/0052/09). Letter of support was obtained from Tigray Regional Health Bureau and the selected districts health offices. The participants were informed about the purpose of the study and written informed consent was obtained from each study participant before the interview. Confidentiality was assured and participants were informed that they have the right to withdraw from interview at any stage.

## Results

### Socio-demographic characteristics

A total of 68 HEWs participated in the study with a mean age of 30.44(±5.52 SD) years. From the total of 68 HEWs, one-fourth (25%) were with certificate educational level, two-third (66.2%) were currently in union in their marital status and 44(64.7%) of them had received training bout TB within the last 2 years (Table 1).

**Table 1:** Socio-demographic characteristics of the respondents, in Tigray region, Northern Ethiopia, 2018 (N = 68).

Variable	Number	Percent
**Age**		
20–29 years	32	47.1
≥ 30 years	36	52.9
**Marital status**		
Currently not in union*	23	33.8
Currently in union	45	66.2
**Educational status**		
Certificate	17	25
Diploma	51	75
**Received training on TB within the last 2 years**		
Yes	44	64.7
No	24	35.3
**Knowledge of HEWs on TB**		
Good	28	41.2
Poor	40	58.8
**Total work experience**		
≤ 5years	23	33.8
> 5years	45	66.2
**Total population of village on which HEWs served**		
≤ 2500	21	30.9
2501–5000	13	19.1
>5000	34	50

*(single, widowed and separated).

### Implementation of CBTC activities

Three-fourth (77.9%) of HEWs had annual plan on CBTC activities and 62(91.2%) had provided health education about TB to communities in the last three months. Two-third (66.4%) and 31(45.5%) of HEWs had conducted TB screening at communities level and TB household contacts respectively. Of the total 68 HEWs, 39(57.4%) had identified and referred at least one presumptive TB and 31(45.6%) of them had provided CB-DOTS service. About 42(61.8%) of HEWs had involved community volunteers (WDA/kebele leader) at different CBTC activities (Table 2).

**Table 2:** Implementation of CBTC activities, in Tigray region, Northern Ethiopia, 2018 (N = 68).

Variable	Number	Percent
**HEWs have integrated TB plan with annual plan**		
**Yes**	53	77.9
**No**	15	22.1
**HEWs have provided health education on TB to the community in the last 3 months**		
Yes	62	91.2
No	6	8.8
**HEWs have conducted TB screening in the community**		
Yes	44	64.7
No	24	35.3
**HEWs have conducted TB screening to HH contacts**		
Yes	31	45.6
No	37	54.4
**HEWs who have identified and referred at least one presumptive TB**		
No	39	57.4
Yes	29	42.6
**HEWs have provided community DOTS service for TB cases**		
Yes	31	45.6
No	37	54.4
**Involvement of community volunteers on CBT Cactivities**		
Yes	42	61.8
No	26	38.2
**HEWs who received supportive supervision on CBTC in the last 6 months**		
Yes	37	54.4
No	31	15.6
**HEWs have communication with PHCU TB focal person on CBTC activities**		
Yes	55	80.9
No	13	19.1

Most of the FGDs participants explained that WDA leaders’ participation has a great role in the presumptive TB identification and referral by HEWs. They increase the chance of identifying presumptive TB cases and promote referral to the communities in which they dwell. Receiving health education from HEWs was the most mentioned reason to search presumptive TB within their team. WDA leaders are not only helping to HEWs by notifying presumptive TB but also by referring such cases to them.

*“We help HEWs in notifying people with TB symptoms like cough for two or more weeks*, *for which they could not get them easily but we can, and many individuals are referred by HEWs from such of our identification”(FGD participant)*.

*“I myself made house to hose visit to many households and there was a girl who was very fatty before a time and becomes emaciated through time*. *After that I informed a HEW and went together during which time the HEW convinced and took her to health facility and was diagnosed TB for which she is taking her medication currently” (FGD participant)*.

The WDA leaders indicated that most of community members accept and obey with their advice. However, there are some community members who don’t want an advice from the WDA leaders for different reasons like lack of confidence on the WDA leader’s capacity and trust to keep their confidentiality. Some FGDs participants noted that, some community members prefer to go to the health facility for diagnosis by themselves to maintain their confidentiality.

*“There are some persons who joke on us and even insult us saying that “go there*! *Take care of your health first*, *we do not want your help*, *we ourselves know better about our health” and sometimes they even laugh on us*. *Additionally there are some people who do not accept our advice but utilize the service in health facilities*. *When we asked such people why they refuse our advice*, *for which they use it*, *they said that they had not trust on us in keeping their confidential issues*. *Some WDA leaders are also provoke for this problem as we expose confidential issues for which we loss acceptance from the communities” (FGD participant)*.

#### HEWs contribution on TB case notification

**Fig 1:**
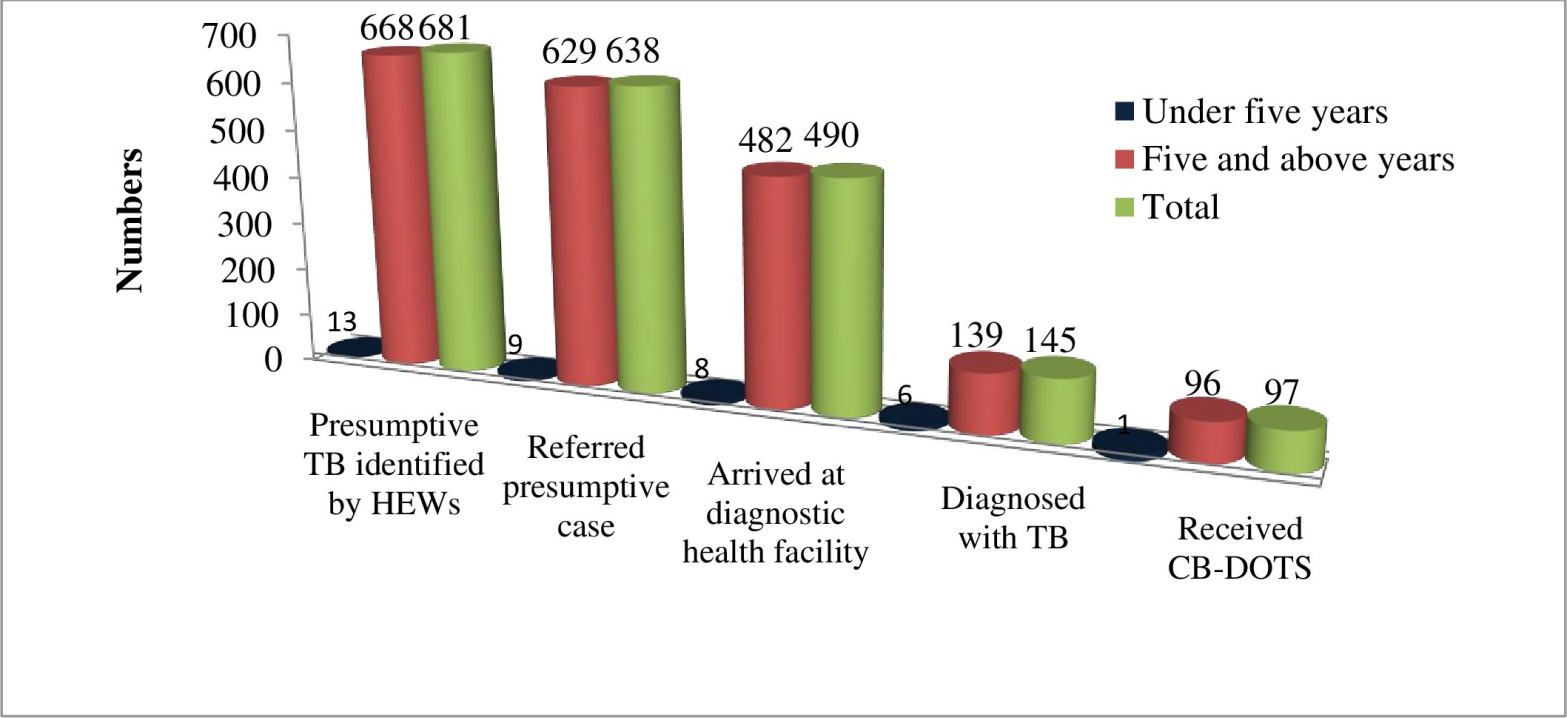
Number of presumptive TB referred by HEWs to diagnosis health facilities in Tigray region, Northern Ethiopia, From March 1, 2017 to February 28, 2018.

From March 1, 2017 to February 28, 2018, a total of 638 presumptive TB cases were referred to diagnostic health facilities by HEWs, and 490(76.8%) of them were arrived at diagnostic health facilities for further investigation. Among those who arrived for investigation, 145 (29.6%) were diagnosed with TB (all forms) (Fig 1).

**Fig 2:**
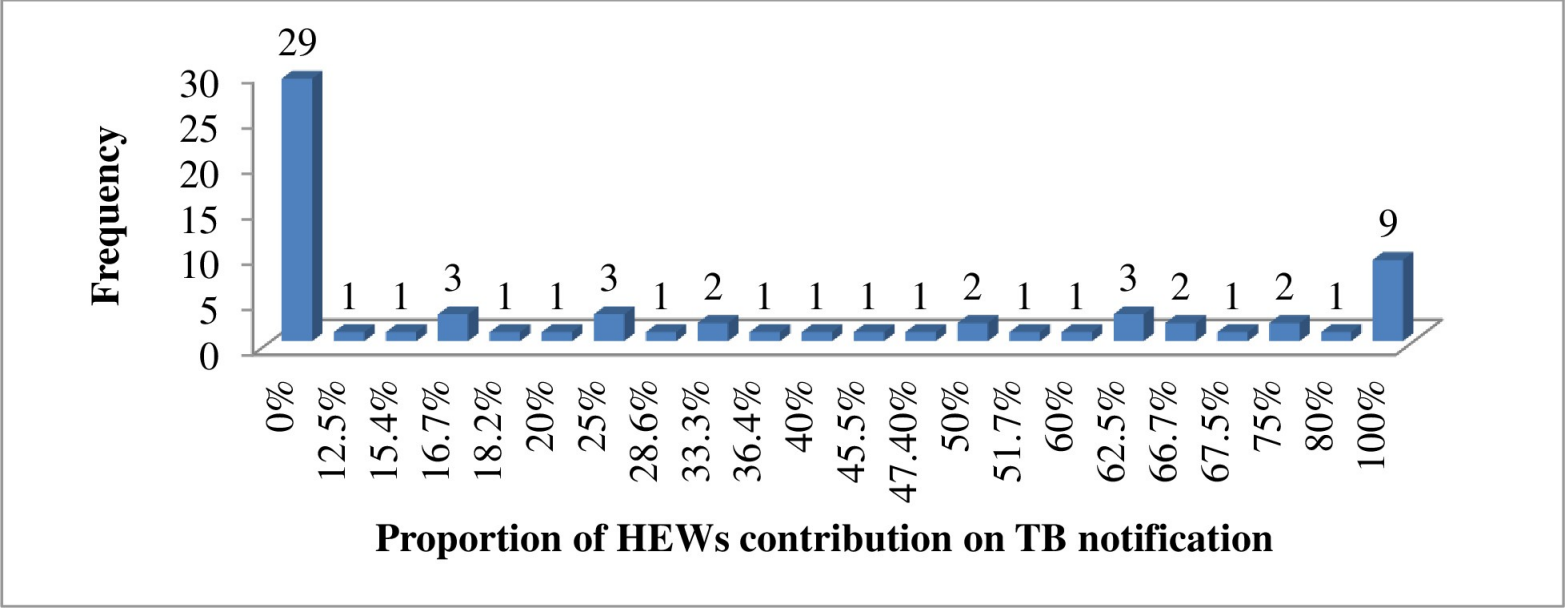
Proportion of HEWs contribution on TB case notification in Tigray region, Northern Ethiopia, from March 1, 2017 to February 28, 2018 (N = 68).

During the specified reporting period, a total 427 TB cases notified in the study areas and 34% (145/427) of them were notified by the contribution of HEWs referral. Of the total 68 HEWs, 39 (57.4%) were contributed at least one TB case and their contribution was ranged from 0% (n = 29) to100% (n = 9) (Fig 2).

#### Presumptive TB identification and referral system

Most KII participants indicated that HEWs have conducted presumptive TB cases identification and referral during routine house to house visit, community meeting/gathering, TB contacts tracing and consultation by WDA leaders. Most of the time, HEWs could not cover the whole community to identify the presumptive TB cases through the routine house to house visit due to the high workload of the 16 HEP packages (for instance: immunization, family planning, antenatal care follow-up and other curative activities). However, they use WDA leaders to facilitate the presumptive TB identification and referral in their kebeles. WDA leaders notify to HEWs if they identify presumptive TB in their team during house to house visit and receiving information from their networks. Then after HEWs visit the household of presumptive TB during which they decide to refer him/her to nearest diagnostic health facility for further investigation. As most of the KII participants indicated that in most cases HEWs used referral paper for referral linkage of the presumptive TB and received feedback from the diagnostic health facilities. However, there were complain on the referral linkage of presumptive TB cases when referred presumptive TB arrived at the diagnostic health facility because all presumptive TB cases were not reached to TB focal person rather they went to card room and out-patient department as other clients. Due to this reason, some presumptive TB dissatisfied because of the payment for investigation card and examination, and unnecessary delay. Additionally, HEWs did not receive referral feedback and failure to register for some presumptive TB cases. Most of KII participants recommended the importance of triaging at entry point of diagnostic health facilities to link the referral of presumptive TB cases by HEWs to TB focal person.

#### Factors associated with HEWs contributions on TB case notification

In a multivariable analysis, CB-DOTS and involvement of community volunteers on CBTC activities were significantly associated with HEWs contributions on TB case notification. HEWs who had provided CB-DOTS were almost four times more likely to have contribution on TB case notification than those HEWs who had not provided CB-DOTS (AOR = 3.63, 95% CI = 1.18–11.19). HEWs who had involved community volunteers on CBTC activities were three times more likely to have contribution on TB case notification than those HEWs who had not community involvement (AOR = 3.31, 95% CI = 1.10–10.09) (Table 3).

**Table 3:** Logistic regression analysis of selected variables with HEWs contributions on TB case notification in Tigray region, Northern Ethiopian, 2019 (N = 68).

Variables	HEWs contribution on TB case notification		COR (95% CI)	AOR) (95% CI)
	No (%)	Yes (%)		
**Having integrated TB plan with annual plan**				
Yes	20(37.7)	33(62.3)	2.46(0.77–8.00)	1.57(0.41–6.05)
No	9(60)	6(40)	1	1
**Conducting TB screening in the community**				
Yes	16(36.4)	28(63.6)	2.07(0.75–5.68)	1.14(0.32–4.09)
No	13(54.2)	11(45.8)	1	1
**Providing community CB-DOTS**				
Yes	7(22.6)	24(77.4)	**5.03(1.73–14.62)** **	3.63(1.18–11.19)*
No	22(59.5)	15(40.5)	1	1
**Involvement of community volunteers on CBTC activities**				
Yes	12(28.6)	30(71.4)	**4.72(1.65–13.49)** **	**3.31(1.10–10.09)** *
No	17(65.4)	9(34.6)	1	1
**Availability of presumptive TB referral paper**				
Yes	24(39.3)	37(60.7)	3.85(0.69–21.49)	1.16(0.15–9.23)
No	5(71.4)	2(28.6)	1	1
**Availability of standard monthly CBTC reporting format**				
Yes	12(30.8)	27(69.2)	**3.19(1.17–8.70)** *	2.01(0.65–6.15)
No	17(58.6)	12(41.2)	1	1
**Availability of CBTC guideline**				
Yes	4(28.6)	10(71.4)	2.16(0.60–7.73)	1.60(0.36–7.04)
No	25(46.3)	29(53.7)	1	1

*statistically significant at 0.05<p<0.01

** statistically significant at 0.01< p<0.001

*** statistically significant at p<0.001.

The qualitative findings indicated that high workload of HEWs, inaccessibility of TB diagnostic services at nearby health facilities, and transportation and investigation costs were identified as factors affecting for presumptive TB referral by HEWs.

*“We have not a lab technician in our nearest health center and some people refuse to go to other diagnostic health facilities by complaining for transportation cost and they went to other religious places like holly water*”*(HEW,KII)*.

## Discussion

This study aims to describe the contribution of HEWs on TB case notification and its associated factors in Tigray region, Northern Ethiopia. This study revealed that 34% of TB cases were notified by HEWs referral. The current finding is almost consistent with previous reported from Ethiopia, 38% of TB cases were referred by HEWs [14]. However, it is higher than reported from Somali Region, Ethiopia (20.3%) of pulmonary TB patients were referred by HEWs [20], and in Nigeria (15.5%) of TB cases were diagnosed for TB by the contribution of community health workers [21]. The discrepancy could be due to the difference in study methods and periods.

The result of this study indicated that the contribution of HEWs on TB case notification was low as only one-third of TB cases were referred by HEWs. This is not in line with the national TB programme strategy wherein HEWs have been given the responsibility to identify and refer all presumptive TB cases in their serving kebeles [13]. This low contribution of HEWs on TB case notification could be due to the high workload of HEWs. HEWs have high workload because they are engaged in 16 HEP packages and therefore could not give all their time to identification and referral of presumptive TB. HEWs are more focused on maternal and child health and they spend their major time on reproductive, maternal, newborn, and child health activities [22]. Furthermore, the qualitative finding highlighted that inaccessibility of TB diagnostic services at nearby health facilities, and transportation and investigation costs were factors affecting for presumptive TB referral by HEWs. This finding is consistent with previous studies which reported that lack of TB diagnosis service and indirect costs were barriers for TB case finding [23–26]. These factors might be reasons for the presumptive TB case to choose traditional medicines over the health facilities and hence this could be possible causes for the low contribution of HEWs on TB case notification.

Our finding show that HEWs who had provided CB–DOTS at health post were more likely to have contribution on TB notification than those HEWs who had not provided CB-DOTS. This finding is supported with previous studies which revealed that CB-DOTS increasingly promoted as an intervention to improve TB case detection and treatment adherence as community health workers are familiar with the layout of community and increases community’s trust [27,28]. CB-DOTS implementation supports community members to be involved in developing local solutions to increase TB case notification [29]. In addition, CB-DOTS helps to increase access to quality TB services [30], and each DOTS session is used as an opportunity for TB contact screening and providing health education on adherence, diagnosis and, TB prevention and control [31]. This could result to improve communities’ health seeking behavior for TB diagnosis and increase awareness on TB.

Community members help to bridge gaps between health system and community through support and coordination [27]. Previous studies from elsewhere revealed that participation of community members had vital role to improve TB case notification [31–35]. In the current study, HEWs who involved community volunteers on CBTC activities were more likely to have contribution on TB case notification compared to those who did not involve community volunteers on CBTC activities. This could be due to the active involvement of community members mainly WDA leaders in presumptive TB identification. This finding is supported by the previous studies done in Ethiopia which revealed that HEWs who discussed TB issues through regular meeting with WDA leaders had a role in improving TB screening practice [19]. In Ethiopia, the WDA leaders are primary contact with communities and they are expected to search for people with symptoms of TB in their team by asking whether anyone in the households have had a cough for two weeks or more and then they inform to HEWs immediately if there are symptomatic TB individuals [13]. Furthermore, WDAs supports HEWs in liaising them with community members. HEWs have been conducting regular meetings with the WDA leaders, exchanging feedback on their work and receiving reports of activities performed by the WDA leaders [19,36].

### Limitation of the study

Poor document handling and failure to remember TB cases referred by HEWs might introduce recall bias and underestimate the contribution of HEWs on TB notification. Social desirability bias may also be introduced in the form of over-reporting for those acceptable and under-reporting for those undesirable reports. However, we have validated respondents’ responses through document reviews and observational technique to reduce the possible recall and social desirability biases. Additionally, we have clearly explained purpose of the study to each respondent before starting the interview to reduce social desirability and we have tried to associate our questions with special events during interview to reduce recall bias. Furthermore, the sample size of this study was relatively small which could affect the statistical inferences of the modeling and caution is needed in generalizing of these results.

### Conclusions

Our study revealed that one-third (34%) of TB cases were notified by the contribution of HEWs referral. Provision of CB-DOTS and involvement of community volunteers in CBTC activities should be strengthened to improve the HEWs contribution on TB notification. Additionally, HEWs should be empowered and further interventions of TB diagnostic services at diagnostic health facilities are needed to improve presumptive TB referral by HEWs.

## Supporting information

S1 Fig S1 Fig which was used as schematic presentation of sampling procedure. (TIF)

S1 File This is the S1 File questionnaire which was used to collect a data from HEWs. (PDF)

S2 File This is the S2 File questionnaire which was used to collect a qualitative data from FGD. (PDF)

S3 File This is the S3 File questionnaire which was used to collect a qualitative data from KII. (PDF)

S1 Data Quantitative data. (SAV)

## References

[1] World Health Organization (WHO). Global Tuberculosis Report 2018. Geneva: World Health Organization; 2018.

[2] World Health Organization (WHO). The End TB Strategy, Global strategy and targets for tuberculosis prevention, care and control after 2015. Geneva: World Health Organization; 2014.

[3] Ho Jennifer, Fox GJ Marais BJ. Passive case finding for tuberculosis is not enough. Int J Mycobacteriology 2016; 5(4):374–8. doi: 10.1016/j.ijmyco.2016.09.023 27931676

[4] World Health Organization (WHO). Global Tuberculosis Report 2016. Geneva: World Health Organization; 2016.

[5] World Health Organization (WHO). Community engagement in tuberculosis. Geneva: World Health Organization; 2014.

[6] World Health Organization (WHO). Integrating community-based tuberculosis activities into the work of nongovernmental and other civil society organizations: Operational guidance. World Health Organization; 2012.

[7] Singh D, Negin J, Otim M, Orach CG, Cumming R. The effect of payment and incentives on motivation and focus of community health workers: five case studies from low- and middle-income countries. Hum Resour Health 2015; 13:58. doi: 10.1186/s12960-015-0051-1 26169179 PMC4501095

[8] Federal Democratic Republic of Ethiopia Ministry of Health. Health Sector Strategic Plan (HSDP-III) 2005/6–2009/10; 2005.

[9] Workie NW, Ramana GN V. The health extension program in Ethiopia. The World Bank, Washington DC; 2013.

[10] Federal Democratic Republic of Ethiopia Ministry of Health. Health Sector Transformation Plan (HSTP) 2015/16–2019/20 (2008–2012 EFY); 2015.

[11] Federal Democratic Republic of Ethiopia Ministry of Health. Comprehensive training manual for clinical and programmatic management of TBL and TB/ HIV. Addis Ababa: Ministry of Health; 2017.

[12] Weldemarium TD, Getachew M. Role of Health Extension Worker in Tuberculosis Prevention and Control in Ethiopia: Systematic Review. 2019; 7(1):1–4.

[13] Federal Democratic Republic of Ethiopia Ministry of Health. National Guideline for TBDR-TB and leprosy in Ethiopia sixth ed. Addis Ababa: Ministry of Health; 2017.

[14] Fekadu L, Hanson C, Osberg M, Makayova J, Mingkwan P, Chin D. Increasing Access to Tuberculosis Services in Ethiopia: Findings From a Patient-Pathway Analysis. 2017; 216(7). doi: 10.1093/infdis/jix378 29117346 PMC5853928

[15] Datiko DG, Yassin MA, Tulloch O, Asnake G, Tesema T, Jamal H, et al. Exploring providers ‘ perspectives of a community based TB approach in Southern Ethiopia: implication for community based approaches. BMC Health Services Research 2015 15:501 doi: 10.1186/s12913-015-1149-9 26553340 PMC4638085

[16] Yassin MA, Datiko DG, Tulloch O, Markos P, Aschalew M, Shargie EB, et al. Innovative Community-Based Approaches Doubled Tuberculosis Case Notification and Improve Treatment Outcome in Southern Ethiopia. 2013; 8(5). 10.1371/journal.pone.0063174 PMC3664633 23723975

[17] Datiko DG, Yassin MA, Theobald SJ, Cuevas LE. A community-based isoniazid preventive therapy for the prevention of childhood tuberculosis in Ethiopia. Int J Tuberc Lung Dis. 2017;21(9):1002–7. doi: 10.5588/ijtld.16.0471 28826449 PMC5566998

[18] Datiko DG, Yassin MA, Theobald SJ, et al. Health extension workers improve tuberculosis case finding and treatment outcome in Ethiopia: a large-scale implementation study. BMJ Glob Health 2017; 2:e000390. doi: 10.1136/bmjgh-2017-000390 29209537 PMC5704104

[19] Gebretnsae H, Ayele BG, Hadgu T, Haregot E, Gebremedhin A, Michael E, et al. Implementation status of household contact tuberculosis screening by health extension workers: assessment findings from programme implementation in Tigray region, northern Ethiopia. BMC Health Services Research 2020 20:72 doi: 10.1186/s12913-020-4928-x 32005226 PMC6995142

[20] Getnet F, Hashi A, Mohamud S, Mowlid H, Klinkenberg E. Low contribution of health extension workers in identification of persons with presumptive pulmonary tuberculosis in Ethiopian Somali Region pastoralists. BMC Health Serv Res. 2017; 17:193. doi: 10.1186/s12913-017-2133-3 28284193 PMC5346224

[21] Adejumo AO, Azuogu B, Okorie O, Lawal OM, Onazi OJ, Gidado M, et al. Community referral for presumptive TB in Nigeria: a comparison of four models of active case finding. BMC Public Health 2016; 16:177. doi: 10.1186/s12889-016-2769-7 26905034 PMC4763441

[22] Mangham-jefferies L, Mathewos B, Russell J, Bekele A. How do health extension workers in Ethiopia allocate their time? Human Resources for Health 2014, 12:61. doi: 10.1186/1478-4491-12-61 25315425 PMC4209031

[23] Ereso BM, Yimer SA, Gradmann C, Sagbakken M. Barriers for tuberculosis case finding in Southwest Ethiopia: A qualitative study. PLoS ONE 2020; 15(1): e0226307. doi: 10.1371/journal.pone.0226307 31895932 PMC6939902

[24] Gebreegziabher SB, Yimer SA, Bjune GA. Qualitative Assessment of Challenges in Tuberculosis Control in West Gojjam Zone, Northwest Ethiopia: Health Workers ‘ and Tuberculosis Control Program Coordinators ‘ Perspectives. Hindawi Tuberculosis Research and Treatment; 2016. 10.1155/2016/2036234 PMC4811263 27066271

[25] Alene GD, Yimer SA. Geographic Accessibility, Readiness, and Barriers of Health Facilities to Offer Tuberculosis Services in East Gojjam Zone, Ethiopia: A Convergent Parallel Design. Dove press Research and Reports in Tropical Medicine 2020:11. 10.2147/RRTM.S233052 PMC7007782 32099509

[26] Mohammed H, Oljira L, Teji K, Ngadaya E, Ajeme T. Burden of Tuberculosis and Challenges Related to Screening and Diagnosis in J ClinTuberc Other Mycobact Dis Burden of tuberculosis and challenges related to screening and diagnosis in Ethiopia. J ClinTuberc Other Mycobact Dis 2020; 19. 10.1016/j.jctube.2020.100158 PMC7113623 32258437

[27] Arshad A, Salam RA, Lassi ZS, Das JK, Naqvi I, Bhutta ZA. Community based interventions for the prevention and control of tuberculosisinfectious Diseases of Poverty 2014, 3:27 http://www.idpjournal.com/content/3/1/27. 10.1186/2049-9957-3-27 PMC4136404 25136445

[28] U.S. Agency for International Development. Accelerating Impact: Expanding Access to Care U.S. Government Report on International Foreign Assistance for TB FY 2011/2012. Washington, DC; 2012.

[29] Ahmad B, Qader G, Rashidi MK. Community-based DOTS for improved TB case detection and treatment outcomes in Afghanistan. Technical brief USAID/MSH, Challenge TB; 2019.

[30] Bayly T. TB care I final report 2010–2015; 2015.

[31] Colvin C, Mugyabuso J, Munuo G, Lyimo J, Oren E, Mkomwa Z. Evaluation of community-based interventions to improve TB case detection in a rural district of Tanzania. Global Health: Science and Practice 2014; 2 (2). 10.9745/GHSP-D-14-00026 PMC4168621 25276579

[32] Olusola-Falae B, Obeagu EI, Odo M, Ochei KC, Solanke E, Idaboh T. Impact of community based tuberculosis care interventions on TB Case detection in Nigeria–What works and what does not? Int. J. Adv. Multidiscip. Res. 2016; 3(2): 30–39.

[33] Querri A, Ohkado A, Yoshimatsu S, Coprada L, Lopez E, Medina A et.al. Enhancing tuberculosis patient detection and care through community volunteers in the urban poor, The Philippines. International Union against Tuberculosis and Lung Disease Health solutions for the poor, Public Health Action 2017; 7(4). 10.5588/pha.17.0036 PMC5753779 29584799

[34] Makombe R, Soto A, Abdula A, Miller C, Conjera J, Cadir N et.al. Implementation brief: interventions in Cambodia, Mozambique & Myanmar; 2018.

[35] Ayu IG, Desy P, Jauhar M, Rachmawati U, Kusumawardani LH. Empowering community health volunteer on community- based tuberculosis case management programs in lower- income countries: A systematic review. Journal of Community Empowerment for Health 2019;2(2):172–80.

[36] Kok MC, Kea AZ, Datiko DG, Broerse JEW, Dieleman M, Taegtmeyer M, et al. A qualitative assessment of health extension workers ‘ relationships with the community and health sector in Ethiopia: opportunities for enhancing maternal health performance. Human Resources for Health 2015; 13:80 doi: 10.1186/s12960-015-0077-4 26423049 PMC4589131

