# Supplementary figures and images for: Health extension workers contribution on tuberculosis case notification in Tigray region, Northern Ethiopia: A concurrent mixed method study
Source: PLoS One. 2022 Aug 16;17(8):e0271968. doi: 10.1371/journal.pone.0271968 (PMC9380935; doi:10.1371/journal.pone.0271968)

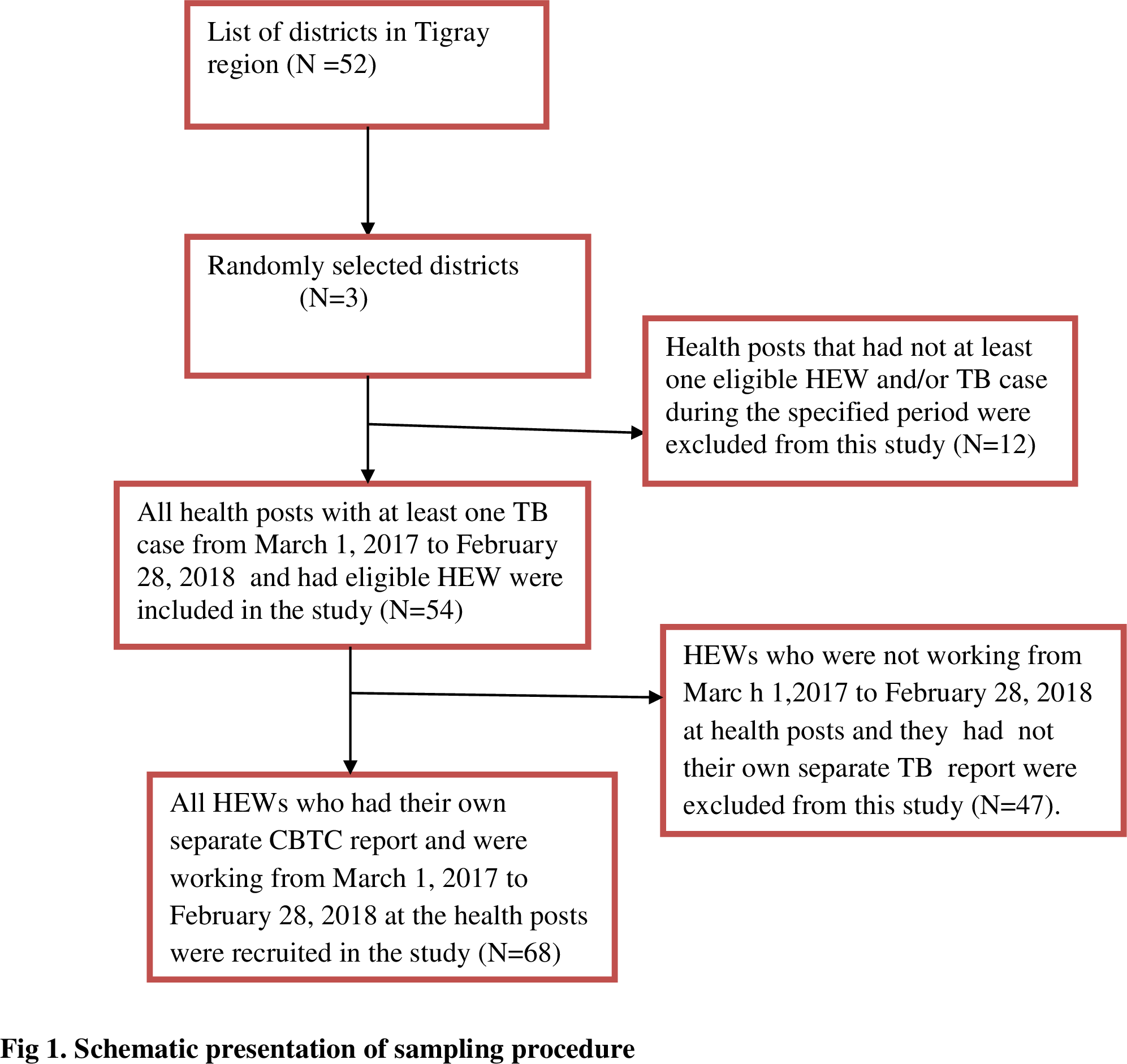

Supplement: S1 Fig — (TIF) [file pone.0271968.s001.tif]
