# Supplementary material for: Health extension workers contribution on tuberculosis case notification in Tigray region, Northern Ethiopia: A concurrent mixed method study
Source: PLoS One. 2022 Aug 16;17(8):e0271968. doi: 10.1371/journal.pone.0271968 (PMC9380935; doi:10.1371/journal.pone.0271968)
Supplement: S1 File — (PDF) [file pone.0271968.s002.pdf]

Health post/ HEWs Questionnaire

Information sheet and consent form

Name of Worreda …………………Name of health post ....Name of HC…………….

Name of Kebele .... Name of serving Kushet/Got (listed all serving kushet and Got)…………………………….. Questionnaire identification number..........................

Information sheet

Hello! My name is ______________ I am a member of a research team. A research on TB is conducting by Tigray health research institute in collaboration with TB challenge on research title: Health extension workers contribution on tuberculosis cases notification in Tigray region, northern Ethiopian. The objective of this study is to identify the contribution of HEWs on TB notification cases and its associated factors. I am one of the data collectors and I am going to ask you some questions about socio-demographic, knowledge of HEWs on TB, implementation of CBTC activities, availability and utilization of TB related reporting and recording tools. Your name will not be written in this form and will never be used in connection with any of information you tell me. However, your honest answers to these questions will help to solving the problem on tuberculosis cases notification and factors affecting presumptive TB identification and referral by HEWs in Tigray Region. The interview will take about 1hour and we are appreciating your help in responding to this research questions .You have a right not to participate and withdraw at any time of interview.

Would you be willing to participate? Yes……., No……

Consent form

I have been briefly informed about the study and clearly understood the objective of the study. So I am agreeing to participate in this study.

Signature ………………………

Result

Result codes: Completed=1, Refused=2, partially completed=3, respondent no found=4

Name of interviewer ………………………..signature………………..date……………….

Interviewer code……………………..

Name of supervisor …………………………signature………………...date………………

Annex II: Questionnaire for Health post(HEW)

Part I: General Information		Response			Skip	Remark
101	Population size		Total Kebele	Serving catchment by HEW		
		5 and above				
		Under five				
		Total				
102	Household size					
103	Number of WDG					
104	Number of HEW in your kebele	…………(Number)				
105	Age of HEW in complete years (interviewee)	………….(Years)				
106	Marital status of HEW	1.Single<br>2. Married<br>3. Divorce<br>4. Widowed<br>5. Separated<br>99. Other (Specify)...........				
107	Educational level of HEW?	1.Certificate<br>2. Diploma(HEW)<br>3. Diploma(Nurse)<br>99. Other (Specify)...........				
108	Training status of HEW on TB in last 2 years?	1.Trained<br>2.Not Trained<br>98. I don't remember				
109	Work experience in complete years?	……… (Year)				
**Part II. HEWs Knowledge on TB**						
201	Do you know the cause of TB?	1.Yes<br>2. No			If 2 Skip to Q203	
202	If yes to Q201, could you tell me the causes please?	1. Cold weather<br>2. Unstable weather condition<br>3. Mycobacterium tuberculosis bacteria				

		4. Pneumococcal bacteria<br>5. Fungus<br>99. Others (Specify)........		
203	Do you know the mode of transmission of TB?	1.Yes<br>2. No	If 2 Skip to Q205	
204	If yes to Q203, what is/are the mode/s of transmission?	1. Through air<br>2. Blood contact<br>3. Contaminated food and water<br>99. Others (Specify)............		
205	Do you know the cardinal symptoms of TB? (**More than one response possible**)	1.Cough of ≥ two weeks<br>2. Night sweating<br>3. Loss of weight<br>4. Fever<br>5. Loss of appetite<br>99. Others (Specify)...........<br>98. I don't know		
206	What are the TB diagnostic methods? **(More than one response possible)**	1.Sputum examination by AFB<br>2.Gen x-pert<br>3. Radiology<br>4. Culture<br>5. Blood examination<br>6. Cytology/Pathology<br>98. I don't know<br>99. Others (Specify)..........		
207	What are the targeted groups for systematic screening of active TB? **(More than one response possible)**	1. Contact of TB patient<br>2. People living with HIV<br>3. Diabetes mellitus patients<br>4. Mal nutrition persons<br>5. People working in industries<br>99. Others (Specify)..........<br>98. I don't know		
**Part III. HEWs Practices on TB prevention activities**				
301	Does the health post have annual plan for 2010 EFY? (**check any**	1. Yes<br>2. No	If 2 Skip to 303	

	**displayed or documented plan)**			
302	If yes to Q301, is TB plan integrated in the annual plan of HEW? (Community Education, TB screening and referral, DOTS)	1. Yes<br>2. No		
303	Do you monitor TB prevention and control activities based on achievment VS plan?	1. Yes<br>2. No	If 2 Skip to 305	
304	If yes to Q 303, frequency of monitoring?	1. Every month<br>2. Every Quarter<br>3. Every 6th month<br>4. At the end of year<br>99. Others (Specify)............		
305	Do you fill thedecument performance chart abou TB prevention activity performances in the last one year?(See the document chart)	1. Yes<br>2. No		
306	Do you give health education on TB to the community in the last 3 month?(check documents)	1. Yes, supported with documents<br>2. Yes, not supported by document<br>3. No	If 3 Skip to 308	
307	If yes to Q306, what approaches do you use?<br>(More than one response possible)	1. House to house visit<br>2. Educating at Health post<br>3. Using WDGL<br>4. Through community gathering<br>99. Other (Specify)…………		
308	Expected number of presumptive TB in your kebele? (Last one year)?	……….(Number)		
309	Did you conduct screening for TB in your kebelle? (Check any records)	1. Yes<br>2. No	If 2 Skip to Q311	
310	If Yes to Q309, what approaches are using for TB screening?	1.Home to home<br>2. Health post		

	(More than one response possible)	3. Using WDG<br>4. Outreach services<br>5. Community gathering<br>99. Others (Specify)........				
311	Are HEWs practicing TB screening for HP visitors? (check , and document the proportion of TB screening done to HP visitors in last 1 month)	1. Yes<br>2. No<br>If yes, %..........<br>(number of screened/number of visitors)				
312	Did HEWs identify presumptive TB in your kebelle? (Check records & document finding)	1.Yes<br>2. No			If 2 Skip to 317	
313	If yes to 312, number presumptive TB identified in your kebell? (Last one year)?		Kebele	Serving catchment		
		5 and above				
		Under five				
		Total				
314	Total number presumptive TB referred to HF for investigation? (Last one year)?	5 and above				
		Under five				
		Total				
315	How many of the referred presumptive TB reached health facility (last one year)?	5 and above				
		Under five				
		Total				
316	Number TB cases diagnosed from the presumptive TB cases (last one year)?	5 and above				
		Under five				
		Total				
317	Do you practice house hold /Close contacts screened for TB? (Check records, and document)	1. Yes<br>2. No			If 2 Skip to Q327	
318	If yes to Q317, number of TB index cases whose house holdcontacts screened for TB in the last one year?		Kebele	Serving catchment		
		5 and above				
		Under five				
		Total				

319	If yes to Q317, number of House hold/ close contacts screened in the last one year?	5 and above				
		Under five				
		Total				
320	Number of presumptive TB identified among house hold /close contacts (last one year)?	5 and above				
		Under five				
		Total				
321	Number presumptive TB referred to HF for diagnosis among house hold/ close contacts (Last one year)?	5 and above				
		Under five				
		Total				
322	Number of the referred presumptive TB reached health facility among the house hold /close contacts (last one year)?	5 and above				
		Under five				
		Total				
323	Number diagnosed with TB among house hold/close contacts (last one year)?	5 and above				
		Under five				
		Total				
324	Number under five years put on IPT among house hold/close contacts (last one year)?					
325	Does the HF provide you feedback on the presumptive TB refereed from Your HP?(Oral or written feedback)	1. Yes<br>2. No<br>98. Don’t remember				
326	If yes to Q325, number feedback available among presumptive TB referred to nearby HC (last one year)		Kebele	Serving catchment		
		5 and above				
		Under five				
		Total				
327	Number of TB cases expected in the last one year?		………	………		
328	Total number of TB cases diagnosed in your Kebele in the	5 and above				
		Under five				

	last one year?	Total				
329	Do you give DOTS service for TB clients?(Check records and document)	1.Yes<br>2. No			If 2 Skip to Q401	
330	If yes to Q329, number TB case on DOTs in the last one year among diagnosed?	5 and above				
		Under five				
		Total				
331	If yes to Q329, number TB cases on DOTs Currently?	5 and above				
		Under five				
		Total				
**Part IV. HEWs Communication with WDA and PHCU pertaining to TB Program**						
401	How do you communicate with WDG leaders?<br>(Multiple response Possible)	1.Through report<br>2. Meeting<br>3. Home to home visit<br>4. Telephone<br>99. Others (Specify)........				
402	Do you have regular meeting with WDG?	1. Yes<br>2. No			If 2 Skip to Q404	
403	If yes to Q402, frequency of meeting with WDG	1. Biweekly<br>2. Monthly<br>3. Every 3 months<br>4. Every 6 months<br>99. Others (Specify)........				
404	Do you discuss on TB related issues with WDG(check any documented minutes and records)	1. Yes<br>2. No				
405	Have you been supervised by HC staff on TB Program in the last six months?(Program specific and or integrated: Check documented feedback)	1. Yes<br>2. No			If 2 Skip to Q407	
406	If yes to Q405, how many times did you get the SS support?	………..(Number)				
407	Do you have communication with	1. Yes			If 2 Skip	

	the TB focal person in the Catchment health Center?	2. No	to Q409	
408	If yes to Q 407, How do you communicate with TB focal person?<br>(Multiple response Possible)	1. Telephone<br>2. Using the index case (orally)<br>3. Written feedback slip<br>4. Supervisor/other staff<br>99. Others (Specify)...........		
409	Does the WDA/kebele leader have active involvement in TB Program?	1. Yes<br>2. No	If 2 Skip to Q501	
410	If yes to Q 409, what is the type of involvement of the WDA/kebele leader in TB Program?<br>(multiple response possible)	1.TB Community Sensitization<br>2. Mobilizing for presumptive TB screening and referral<br>3. Regular monitoring and review of TB performance<br>4. Mobilizing financial support for TB and related activities<br>5. Mobilizing WDGL and other Kebele actors to engage in TB program<br>99. Other (Specify)…………		
**Part V. Availability and utilization of TB related R& R tools and job aids**				
501	Is TB screening tool available?<br>**(check by observation)**	1. Yes<br>2. No		
502	Is the presumptive TB log book available? (if no, comment on where do presumptive TB recorded)	1. Yes<br>2. No	If 2 Skip to Q504	
503	If yes to Q 502, is the presumptive TB log book is utilized?<br>**(check by observation)**	1. Yes<br>2. No		
504	Is the presumptive TB referral paper available?<br>**(check by observation)**	1. Yes<br>2. No		
505	Is contacts investigation logbook	1. Yes	If 2 Skip	

	available? (if no, comment on where CI recorded)	2. No	to Q507	
506	If yes to Q 505, is contacts investigation logbook is utilized? **(check by observation)**	1. Yes<br>2. No		
507	Is monthly TB reporting format available?	1. Yes<br>2. No		
506	Availability of manuals and IEC/ BCC materials (Multiple response Possible)<br>(**please check by observation)**)	1.TB Pocket guideline (Local language Version)<br>2. TB screening tool<br>3. Integrated Refresher Training (IRT) Manual on TBL<br>4. Community TB care guideline<br>5. TTS card<br>6. TB related posters<br>7.TB related Brochures/Booklets<br>8.TB related Flipcharts<br>9.TB related audio-Visual materials		

Thank you very much!!

ናይ ጥዕና ኬላ/ (ጥ/ጥ ቤተ ሰብ ሰራሕተኛ) መሕትት ቅጥዒ ሓበሬታመውሃቢን ዉዕልስምምዕነትን

ሽም ወረዳ ....ሽም ጥ/ኬላ.............................ሽም ጣብያ....................

ሽም መማእከሊ ጥ/ጣብያ ....ሽም እታ ጥ/ጥ/ሰራሕተኛ እተማኸሎ ቑሸት/ጎጥ

(ኩሎም ቁሸትን ጎጥን ይፀሓፍ) ....ቃለ መሕተት መፍለዪ ቁፅሪ.......................

መእተዊ፡-

ጥዕና ይሃበለይ! ሽ·መይ............. ይበሃል ኢንስቲትዩት ምርምር ጥዕና ትግራይ ምስ ክልል ጥዕና ቢሮን challenge TBን ብምትሕብባር ብዛዕባ ኩነታት ኣተገባብራን ምክንያታትን ብጥርጡራት ንሕማም ዓባይ ሰዓል ዳህሳስን ሪፈር ምባልን ብሰራሕተኛ ጥሙር ጥዕና ኣብ ክልል ትግራይ፣ ሰሜን ኢትዮጵያ ንዘካይድዎ መፅናዕቲ ሓበሬታ ንምእካብ እየ መፂአ፡፡ እዚ መፅናዕቲ ኣተገባብራን ምክንያታትን ጥርጡራት ንሕማም ዓባይ ሰዓል ዳህሳስን ሪፈር ምባልን ንምፍላጥ ዝዓለመ እዩ። ኣነ ካፍቶም መረዳእታ ዝእክቡ ሓደ እየ፣ ስለዚ ንስኹን ኣብዚ ንክትሳተፉ ተሓሪኹን ስለ ዝኮንክን ሓደ ሓደ ሕቶታት ብዛዕባ ሓፈሻዊ ንጥርጡራት ንሕማም ዓባይ ሰዓል ዝግበር ዳህሳስን ሪፈር ምባልን ዝተተሓሓዘ ናይ ባዕልክንን ጥዕና ኬላክንን ኩነታት ዝምልከት ክሓተክን እየ። ንትህባና ሓበሬታ ኩሉ ምስጢሩ ዝተሓለወ እዩ ካብ ንፅንዓት ሓሊፉ ንካሊእ ጉዳይ ዝትሓሓዝ የብሉን፡፡ ናትክን ቅነቀዕና ዝተመለአ ኣብዚ መፅናዕቲ ምስታፍ ኣብ ክልል ትግራይ ዘሎ ናይ ኣተገባብራን ምክንያታትን ንጥርጡራት ንሕማም ዓባይ ሰዓል ዝግበር ዳህሳስን ሪፈር ምባልን ብሰራሕተኛ ጥሙር ጥዕና ዘሎ ፀገም ንምፍላጥ ወሳኒ እዩ። እዚ ድማ ብተዘዋዋሪ ኣብ ሕብረተሰብክን ፀገም ኣብ ምፍታሕ እጃምክን ኣወፊክን ማለት እዩ። እዚ መጠየቅ ብማእኸላይ ሓደ ሰዓት ዝውድእ እንትኮን ብድልየት ዝተመስረተ እዩ፣ ዘይምስታፍ እውን ትክእላ ኢክን ብተወሳኺ ክትምልሰኦ ዘይትድልዮኦ ሕቶ እንትትረከባ እንተለክን ምዝላልንን ኣብ መንጎ እውን ኣብ ዝደለክንኦ ግዘ ምቁራፅ ትክእላ ኢክን። ኣብዚ መፅናቲ ንምስታፍ ፍቓድኛ ዲኹን ? እወ......... ኣይኮንኩን..............

ዉዕል ስምምዕነት ቅጥዒ

ኣነ በቲ ዝተውሃበኒ ሓበሬታ መሰረት ብዛዕባ ዕላማ ናይቲ መፅናዕቲ ተርዲኡኒ ስለ ዝኮነ ኣብቲ መፅናዕቲ ንክሳተፍ ዝተስማዕማዕኩ ምካነይ ብፊርማይ የረጋግፅ፡፡ፊርማ......................................

ውፅኢት እዚ ቃለ መሕተት 1.ዝተመለአ 2.ንምስታፍ ድሌት ዘይብላ/ሉ 3.ብክፉል ዝተመለአ 4.ተሳታፊት ኣይተረከበትን

ሽም ሓታቲ...ፊርማ.................ዕለት............

መፍለዪ ቑፅሪ ሓታቲ......................................

ዘረጋገፀ ሽም ስፖርቫይዘር ....ፊርማ..................ዕለት...

ሕዛል 2፡ መጠይቕ ኣብ ጥዕና ኬላ/ ጥ/ጥ ቤተ ሰብ ሰራሕተኛ ዝእከብ ሓበሬታ

1ይክፋል:ሕቶታትብዛዕባ ሓፈሻዊ ኩነታት						
ተ.ቑ	ዝርዝር ሕቶታት	መማረፂ መልስታት			ዝለል	መብርሂ
101	በዝሒ ህዝቢ		ብጣብያ ደረጃ	ጥ/ጥ/ሰራሕኛ ተማእኽሎም		
		5ተን ልዕሊኡን				
		ትሕቲ 5 ዓመት				
		ድምር				
102	በዝሒ መ/ስድራ?					
103	በዝሒ ል/ጉጅለ?					
104	ጠቅላላ በዝሒ ጥሙር ጥዕና ሰራሕታኛ?	…………(ብቁፅሪ)				
105	ክንደይዕድመክን/ኪ?<br>(ንተጥያቂት ጥ/ጥዕና)	……………..ዓመት)				
106	ኩነታትሓዳርክን/ ኪ?<br>(ንተጠያቂት ጥ/ጥዕና)	1.ዘይተመርዐወት<br>2. ባዓልቲ ሓዳር<br>3. ዝተፋተሐት<br>4. ሰብኣያ ዝሞታ<br>5. ተፋላልዮም ዝነብሩ<br>99. ካሊእ (ይገለፅ)……….				
107	ናይት/ቲደረጃክን /ኪ?<br>(ንተጠያቂት ጥ/ጥዕና)	1.ሰርቲፊኬት<br>2. ዲፕሎማ(ሰ/ጥ/ጥዕና)<br>3. ዲፕሎማ(ነርስ)<br>99. ካሊእ (ይገለፅ)……….				
108	ኣብዚ ውሽጢ ክልተ ዓመት ብዛዕባቲቢ ስልጠናወሲድኪ/ክን ትፈልጢ/ጣ ዶ?	1.እወ<br>2. አይፋሉን				
109	ክንደይ ዓመት ስራሕ ልምዲ ኣለክን/ኣለኪ?	……….ዓመት				
2ይክፋል፡ብዛዕባኣፍልጦሰ/ጥ/ጥዕና ኣብ ምክልካልን ምቁፅፃን ሕማም ቲቢ ዝምልከት ሕቶታት						
201	መንቀሊ ሕማም ቲቢ ትፈልጣ/ ጢ ዶ?	1.እወ<br>2. አይፈልጥን			መልሲ2 →203	
202	ሕቶቁ 201 መልሱእወ እንተኮይ፤መንቀሊኡክትነግር ኒ/ራኒ ትክእላ/ሊ ዶ?	1. ቁሪ/ዝሑል ኣየር<br>2. ምቅይያር ኩነታት ኣየር<br>3. ማይኮባክተርየም ቱበርኩሎሲስ ዝተብሃለ ባክተርያ<br>4. ኒሞኮካል ባክተርያ<br>5. ፋንጋይ				

		99. ካሊእ ይገለፅ..............		
203	መተሓላለፊ መንገዲ ሕማምቲቢ.ትፈልጣ/ ጢ ዶ?	1. እወ<br>2. ኣይፈልጥን	መልሲ2 →205	
204	ሕቶ ቁ 203መልሱ እወ እንተኮይ፤ ብምንታይ ይመሓላለፍ?	1. ብኣየር/ብስርዓተ ምስትንፋስ<br>2. ብደም ንክክእ<br>3. ብዝተበከለ ምግብን ማይን<br>99. ካሊእ ይገለፅ..............		
205	ቀንዲ ምልክታት ሕማምቲቢ እንታይ እንታይን እዮም ?<br>**(ዝዘርዘረቶም ጥራሕ ይከበብ)**	1.ሰዓል ልዕሊ 2 ሰሙን<br>2. ለይቲ ለይቲ ምርሃፅ<br>3. ክብደት ምቅናስ<br>4. ረስኒ<br>5. ናይ ምግቢ ድሌት ምቅናስ<br>99. ካሊእ ይገለፅ..............<br>98. ኣይፈልጦምን		
206	ሕማምቲቢ ብምንታይ ይንፀር/ይፍለጥ?<br>**(ዝዘርዘረቶም ጥራሕ ይከበብ)**	1. ብዓክታ ምርመራ/AFB<br>2. ጂን ኤክስፐርት/Gen x-pert<br>3. ራጂ<br>4. ካልቸር<br>5. ብደም ምርመራ<br>6. ፓቶሎጂ ምርመራ<br>99. ካሊእ ይገለፅ..............<br>98. ኣይፈልጥን		
207	ንፈተሸ ሕማምቲቢ ቅድምያ ዝወሃቦም እንመን እዮም?<br>**(ዝዘርዘረቶም ጥራሕ ይከበብ)**	1. ምስ ሕሙም ቲቢ ዘለዎ ሓቢሮም ዝነብሩ ኣባላት ስድራ ቤት<br>2. HIV ኣብ ደሞም ዘለዎም ሰባት<br>3. ሕማም ሽኮር ዘለዎም ሰባት<br>4. ሕፅረት ምግቢ ዘለዎም ሰባት<br>5. ኣብ ፋብሪካታት ዝሰርሑ ሰባት<br>99. ካሊእ ይገለፅ..............<br>98. ኣይፈልጥን		
**3ይክፋል፡ብዛዕባተግባርሰ/ጥ/ጥዕና ኣብ ምክልካልን ምቁፅፃን ሕማም ቲቢ ዝምልከት ሕቶታት**				
301	ናይ ጥዕና ኬላኩም ዓመታዊ ትልሚ ኣለክን ዶ?(ናይ2010 ዓ/ም ብምረኣይ ይረጋገፅ)	1. እወ<br>2. ኣይፋሉን	መልሲ 2→303	
302	ሕቶ ቁ 301 መልሱ እወ እንተኮይ፤ ኣብቲ ዓመታዊ ትልሚብዛዕባ ምክልካልን ምቁፅፃርን ሕማም ቲቢ ዘካተተዲዩ? (ኣስተምህሮ ጥዕና ሕ/ሰብ፣ዳህሰሳ ሕማም ቲቢ፣ክትትል ሕክምና ሕማም	1. እወ<br>2. ኣይፋሉን		

	ቲቢ.)			
303	ናይ ምክልካልን ምቁፅፃርን ሕማም ቲቢ ስራሕቲ ፍፃመ ምስ ትልሚ ይነፃፀር ዶ? **(ዶከሜንት ረአ)**	1. እወ<br>2. አይፋሉን	መልሲ<br>2→305	
304	ቁ 303 እወ እንተኮይኑ፤ በቢ ክንደይ ይነፃፀር?	1. ወርሓዊ<br>2. በቢ 3 ወርሒ<br>3. በቢ.6 ወርሒ<br>4. ኣብ መወዳእታ ዓመት<br>99. ካሊእ ይገለፅ..............		
305	ስራሕቲ ሕማም ቲቢ ኣብ ናይ ሰ/ጥ/ጥዕና መነፃፀሪ ቻርት ትልምን ፍፃመን ክነፃፀር ይግበር ዶ?	1. እወ<br>2. አይፋሉን		
306	ኣብዚ 3 ወርሒ ውሽጢ ብዛዕባ ቲቢ ዝምልከት ንሕ/ሰብ ኣስተምህሮ ሂብኪ/ሂብክንትፈልጢ/ጣ ዶ? **(ደኩሜንት ረአ)**	1. እወ ብመረዳእታ ዝተደገፈ<br>2. እወ ብመረዳእታ ዘይተደገፈ<br>3. አይፋሉን	መልሲ<br>3→308	
307	ቁ306 መልሱ እወ እንተኮይኑ፤ እንታይ ዓይነት ሜላ ኣተምህሮ ትጥቀሚ? **(ዝዘርዘረቶም ጥራሕ ይከበብ)**	1. ዑደት ገዛ ንገዛ<br>2. ኣብ ጥ/ኬላ<br>3. ብል/ጉጅለ ኣመራርሓ<br>4. ሕ/ሰብ ብምእካብ/ብምስብሳብ<br>99. ካሊእ ይገለፅ...............		
308	ኣብ ጣብያኽን ኣብ ዝሓለፈ ሓደ ዓመት ውሽጢክህልዉ ትፅቢት ዝግበረሎም ንሕማም ዓባይ ሰዓል ዝጥርጠሩ በዝሒ ሰባት ክንደይ እዮም?	…………….(ብቁፅሪ)		
309	ኣብ ጣብያኽን/መማእከሊ ቁሸትክን ፈተሽ ሕማምቲቢ ትገብሪ/ትገብራ ዶ? **(መዝገብ ረአ)**	1. እወ<br>2. አይፋሉን	መልሲ<br>2→311	
310	ቁ 309 መልሱ እወ እንተኮይኑ፤ ንፈተሽ ሕማም ቲቢ እትጥቀማሉ ሜላ እንታይ እዩ? **(ካብ ሓደ ንላዕሊ መልሲ ይካኣል እዩ).**	1. ዑደት ገዛ ንገዛ<br>2. ኣብ ጥ/ኬላ<br>3. ብል/ጉጅለ ኣመራርሓ<br>4. ፈተሽ በፅሕካ ምምላስ<br>5. ሕ/ሰብ ብምእካብ/ብምስብሳብ<br>99. ካሊእ ይገለፅ...............		
311	ናብ ጥዕና-ኬላ ንዝተፈላላዩ	1. እወ		

	ግልጋሎታት ንዝመፁ ተገልገልቲ ናይ ሕማም ዓባይ ሰዓል ዳህሳስ ትገብራ/ሪ ዶ? **(መዝገብ ይረአ)**	2. ኣይፋሉን<br>እወ እንተኮይኑኣብዚ ናይ መወዳእታ ሓደ ወርሒ መመያ ዝተገበረሎም ካብ ዝመፁ ብሚእታዊ የቀምጡ)……….%				
312	ኣብ ጣብያኹን/መማእከሊ ቁሸትኹን ኣብ ዝሓለፈ ሓደ ዓመት ውሽጢ ንሕማም ዓባይ ሰዓልዝተጠርጠሩ ሰባት ኔሮም ዶ?	1. እወ<br>2. ኣይፋሉን			መልሲ 2→317	
313	ቁ 312 መልሱ እወእንተኮይኑ፤ ኣብ ዝሓለፈ ሓደ ዓመት ውሽጢ ንሕማም ዓባይ ሰዓል ዝተጠርጠሩ ሰባት ጠቅላላ በዝሒ ክንደይ እዮም?		ብጣብያ ደረጃ	ጥ/ጥ/ሰራሕኛ ተማእኸሎም		
		5ተን ልዕሊኡን				
		ትሕቲ 5 ዓመት				
		ድምር				
314	ካብቶም ንሕማም ዓባይ ሰዓል ዝተጠርጠሩንምርመራ ናብ ጥ/ትካል ዝተለኣኩ ጠቅላላ በዝሒ ክንደይ እዮም?<br>(ኣብ ዝሓለፈ 1 ዓመት ውሽጢ)	5ተን ልዕሊኡን				
		ትሕቲ 5 ዓመት				
		ድምር				
315	ንሕማም ዓባይ ሰዓልተጠርጢሮም ንምርመራ ካብ ዝተልኣኩ ናብ ጥዕና ትካል ዝበፅሑ ጠቅላላ በዝሒ ክንደይ እዮም? **(ዶኩሜንት ረአ)**<br>(ኣብ ዝሓለፈ 1 ዓመት ውሽጢ)	5ተን ልዕሊኡን				
		ትሕቲ 5 ዓመት				
		ድምር				
316	ናብ ጥዕና ትካል ከይዶም ካብ ዝተመርመሩ ክንደይ ዝኣክሉ ሰባት ሕማም ዓባይ ሰዓል ተረኺብሎም/ተነፂርሎም? **(ዶኩሜንት ረአ)**<br>(ኣብ ዝሓለፈ 1 ዓመት ውሽጢ)	5ተን ልዕሊኡን				
		ትሕቲ 5 ዓመት				
		ድምር				
317	ኣብ ዝሓለፈ ሓደ ዓመት ውሽጢ(ኣብ ዝሓለፈ 1 ዓመት ውሽጢ)ንኣባላት ስድራን ካለኦት ምስ ሕሙም ዓባይ ሰዓል ቅርበት ዘለዎም ሰባትንንሕማም ዓባይ ሰዓል ፈተሽ ተገይርሎም ዶ? **(ሪፖርት ረአ)**	1. እወ<br>2. ኣይፋሉን			መልሲ 2→327	

318	ንሕቶ ቁ 317 መልሱ እወ እንተኮይኑ፣ ክንደይ ዝኣክሉ ሕሙማት ዓባይ ሰዓል (TB index case) ንሓቢሮም ንዝነብሩ ቤተሰቦም /ቅርበት ዘለዎም ሰባት ንሕማም ዓባይ ሰዓል ዳህሳስ ተገይርሎም? (ኣብ ዝሓለፈ 1 ዓመት ውሽጢ)		ብጣብያ ደረጃ	ጥ/ጥ/ሰራሕኛ ተማእኸሎም		
		5ተን ልዕሊኡን				
		ትሕቲ 5 ዓመት				
		ድምር				
319	ንሕቶ ቁ 317 መልሱ እወ እንተኮይኑ፣ ክንደይ ዝኣክሉ ኣባላት ቤተሰብ/ቅርበት ዘለዎም ሰባት ንሕማም ዓባይ ሰዓል ዳህሳስ ተገይርሎም? (ኣብ ዝሓለፈ 1 ዓመት ውሽጢ)	5ተን ልዕሊኡን				
		ትሕቲ 5 ዓመት				
		ድምር				
320	ንሕማም ዓባይ ሰዓል ዳህሰስ ካብ ዝተገበረሎም ኣባላት ቤተሰብ/ቅርበት ዘለዎም ሰባት ክንደይ ዝኣክሉ ንሕማም ዓባይ ሰዓል ተጠርጢሮም ኔሮም? (**ዶኩሜንት ረአ**) (ኣብ ዝሓለፈ 1 ዓመት ውሽጢ)	5ተን ልዕሊኡን				
		ትሕቲ 5 ዓመት				
		ድምር				
321	ንሕማም ዓባይ ሰዓል ካብ ዝተጠርጠሩ ኣባላት ቤተሰብ/ቅርበት ዘለዎም ሰባት ክንደይ ዝኣክሉ ናብ ጥዕና ትካል ሪፈር ተባሂሎም (**ዶኩሜንት ረአ**) (ኣብ ዝሓለፈ 1 ዓመት ውሽጢ)	5ተን ልዕሊኡን				
		ትሕቲ 5 ዓመት				
		ድምር				
322	ንሕማም ዓባይ ሰዓል ተጠርጢሮም ናብ ጥዕና ትካል ካብ ዝተለኣኩ ኣባላት ቤተሰብ/ቅርበት ዘለዎም ሰባት ክንደይ ዝኣክሉ ንምርመራ ናብ ጥዕና ትካል በፂሖም? (**ዶኩሜንት ረአ**) (ኣብ ዝሓለፈ 1 ዓመት ውሽጢ)	5ተን ልዕሊኡን				
		ትሕቲ 5 ዓመት				
		ድምር				
323	ንሕማም ዓባይ ተጠርጢሮም ንምርመራ ካብ ዝተለኣኩ ኣባላት ቤተሰብ/ቅርበት ዘለዎም ሰባት ክንደይ ዝኣክሉ	5ተን ልዕሊኡን				
		ትሕቲ 5 ዓመት				

	ሕማም ዓባይ ሰዓልተረኪብዎም?(**ዶኩሜንት ረአ**)<br>(ኣብ ዝሓለፈ 1 ዓመት ውሽጢ)	ድምር				
324	ካብ ቤተሰብ ሕሙም ዓባይ ሰዓል ትሕቲ 5ተ ዓመት ህፃውንቲ መከላከሊ ሕማም ዓባይ ሰዓል መድሓኒት (IPT) ዝጀምበሩ በዝሒ ህፃውንቲ?<br>(ኣብ ዝሓለፈ 1 ዓመት ውሽጢ)					
325	ናብ ጥዕና ትካል ማእከል መመርመሪ ሕማም ዓባይ ሰዓል ካብዝተልኣኹ ጥርጡራት ሕማም ዓባይ ሰዓል ግብረ መልሲ ይልእኩልኩም ዶ?	1. እወ<br>2. ኣይፋሉን<br>98. ኣይዝክሮ				መልሲ2 →327
326	ንሕቶ ቁ 325 መልሱ እወ እንተኮይኑ፤ ኣብ ዝሓለፈ ሓደ ዓመት ውሽጢ (ኣብ ዝሓለፈ 1 ዓመት ውሽጢ) ካብ ማእከል መመርመሪ ጥዕና ጥካል ሕማም ዓባይ ሰዓል ዝተልኣኽልክን በዝሒ ግብረ መልሲ ክንደይ እዮም?(**ዶኩሜንት ረአ**)		ብጣብያ ደረጃ	ጥ/ጥ/ሰራሕኛ ተማእኽሎም		
		5ተን ልዕሊኡን				
		ትሕቲ 5 ዓመት				
		ድምር				
327	ኣብ ሓደ ዓመት ውሽጢ ክርከቡ ትፅብኢት ዝግበረሎም በዝሒ ሕሙማት ዓባይ ሰዓል?					
328	ኣብ ዝሓለፈ ሓደ ዓመት ውሽጢ (ኣብ ዝሓለፈ 1 ዓመት ውሽጢ) በዝሒ ሕማም ዓባይ ሰዓልዝተነፀረሎም ሕሙማትክንደይ ኔሮም?	5ተን ልዕሊኡን				
		ትሕቲ 5 ዓመት				
		ድምር				
329	ናይ ሕማም ዓባይ ሰዓል መድሓኒት ኣብ ጥዕና ኬላክን ትህባ ዶ? (**መዝገብን ሪፖርትን ይራዩ**)	1. እወ<br>2.ኣይፋሉን			መልሲ 2→401	
330	ኣብ ዝሓለፈ ሓደ ዓመት ውሽጢ(ኣብ ዝሓለፈ 1 ዓመት ውሽጢ)ኣብ ጥዕና		ብጣብያ ደረጃ	ጥ/ጥ/ሰራሕኛ ተማእኽሎም		
		5ተን ልዕሊኡን				

	ኬላክንሕክምና ሕማም ዓባይ ሰዓል ዝተገበረሎምበዝሒ ክንደይ እዮም?	ትሕቲ 5 ዓመት				
		ድምር				
331	ንሕቶ ቁ 329 መልሱ እወ እንተኮይኑ፣ ኣብዚሕዚ እዋን ኣብ ጥዕና ኬላክን ሕክምና ሕማም ዓባይ ሰዓል ዝግበረሎም ዘሎ በዝሒ ክንደይ እዮም?	5ተን ልዕሊኡን				
		ትሕቲ 5 ዓመት				
		ድምር				
**ክፋል IV. ርክብ ጥ/ጥዕና ሰራሕተኛ ምስ ማ/መ/ክ/ጥዕናን ል/ጉጅለን ኣብ ስራሕቲ ሕማም ዓባይሰዓል**						
401	ብከመይ መንገዲ እኺን ምስ ኣመራርሓ ል/ጉጅለ እትራከባ?<br>**(ካብ ሓደ ንላዕሊ መልሲ ይካኣል እዩ)**	1. ብሪፖርት<br>2. ብኣኼባ<br>3. ብዕዶት ገዛ ንገዛ<br>4. ስልኪ ብምድዋል<br>99. ካሊእ (ይግለፅ)............				
402	ምስ ል/ጉጅለ ስሩዕ ኣኼባ ኣለክን ዶ?	1. እወ<br>2. ኣይፋሉን			መልሲ<br>2→404	
403	ንሕቶ ቁ 402 መልሱ እወ እንተኮይኑ፣ በቢክንደይ ግዜ እኺን ምስ ል/ጉጅለ ኣኼባ ትገብራ?	1. ክልተ ሰሙናዊ<br>2. ወርሓዊ<br>3. በቢ 3ተ ወርሒ<br>4. በቢ 6ተ ወርሒ<br>99. ካሊእ እንተሊዩ ይገለፅ..............				
404	ምስ ል/ጉጅለ ኣብ ጉዳይ ሕማም ዓባይ ሰዓል ዘተ/ምይይጥ ትገብራ ዶ?<br>**(ቃለ ጉባኤ ወይ ሪፖርት ረአ)**	1. እወ<br>2. ኣይፋሉን				
405	ኣብዚ 6 ወርሒ ውሽጢ ብናይ ማ/መ/ክ/ጥዕና በዓል ሞያታት ብዛዕባ ሕማም ዓባይ ሰዓል ደገፍን ክትትል ተገይርልክን ዶ ኔሩ?<br>**(ቸክሊ ስትን ግብረ መልስን ብምረኣይ ይረጋገፅ)**	1. እወ<br>2. ኣይፋሉን			መልሲ<br>2→407	
406	ንሕቶ ቁ405 መልሱ እወ እንተኮይኑ፣ ክንደይ ግዜ ደገፍን ክትትል ተገርልኩን?	…………..ግዜ				
407	ምሰ ናይካችመንትኩምጥ/ጣብያ ቲቢፎካል ፐርሰን ርክብ ኣለኩም ዶ?	1. እወ<br>2. ኣይፋሉን			መልሲ<br>2→<br>409	

408	ንሕቶ ቁ 407 መልሱ እወ እንተኮይኑ፣ ርክብኩም ብምንታይ እዩ? **(ካብ ሓደ ንላዕሊ መልሲ ይካኣል እዩ)**	1. ብስልኪ<br>2. መልእክቲ ብሕሙም ዓባይ ሰዓል<br>3. ብፅሑፍ ግብረ መልሲ ወረቀት<br>4.ብሱፐርቫይዘር/ካሊእ በዓል ሞያ<br>99. ካሊእእንተሊዩ ይገለፅ............		
409	ናይ ጣብያኩም ል/ጉጅለ/ ኣማሓዳሪ ኣብ ናይቲቢ ፕሮግራም ተሳትፎ ኣለዎ ዶ?	1. እወ<br>2. ኣይፋሉን	መልሲ<br>2→501	
410	ንሕቶ ቁ 409 መልሱ እወ እንተኮይኑ፣ ኣብ እንታይ እንታይ ስራሕቲ ይሳተፍ? **(ካብ ሓደ ንላዕሊ መልሲ ይካኣል እዩ)**	1. ብዛዕባ ቲቢ ኣብ ሕ/ሰብ ምልዕዓል<br>2. ምልዕዓል ንጥርጡራት ቲቢ ዳህሳስንሪፈር ምባልን<br>3. ናይ ቲቢ ፍፃመ ተከታተሊ ዝኮነ ክትትልን ገምጋምን ምግባር<br>4. ምልዕዓል ገንዘባዊ ድጋፍ ንቲቢን ካልኦት ስራሕትን<br>5. ምልዕዓል ንልምዓት ጉጅለ ኣመራርሓን ካልኦት ናይ ጣብያ ፈፀምቲ ኣካላትን ኣብ ቲቢፕሮግራም ንክሰርሑ<br>99. ካሊእእንተሊዩ ይገለፅ............		
**ክፋል V. ኩነታት ህላወን ምጥቃምን ዝተፈላለዩ ቅጥዕታትን መዛግብትን ሓገዝቲ መምርሒታትን ሕማም ዓባይ ሰዓል**				
501	ናይ ሕማም ዓባይ ሰዓል መፈተሺ ሓጋዛይ ኢደ-መጋብር ኣለክን ዶ? **(ብምርኣይ ይረጋገፅ)**	1. እወ<br>2. ኣይፋሉን		
502	ብሕማም ዓባይ ሰዓል ዝተጠርጠሩ መመዝገቢ መዝገብ ኣለክን ዶ? **(ብምርኣይ ይረጋገፅ)**	1. እወ<br>2. ኣይፋሉን	መልሲ<br>2→504	
503	ንሕቶ ቁ 502 መልሱ እወ እንተኮይኑ፣ እቲ መዝገብ ትጥቀማሉ ዶ? **(ብምርኣይ ይረጋገፅ)**	1. እወ<br>2. ኣይፋሉን		
504	ብሕማም ዓባይ ሰዓል ዝተጠርጠሩ መልኣኪ/ ሪፈራል ወረቀት ኣለክን ዶ? **(ብምርኣይ ይረጋገፅ)**	1. እወ<br>2. ኣይፋሉን		
505	ምስ ሕሙም ዓባይ ሰዓል ሓቢሮም ንዝነብሩቤተሰብ ዳህሳስ /ምስ ተመርመሩ ዝምዝገብሉ መዝገብ ኣለክን ዶ?**(ብምርኣይ ይረጋገፅ)**	1. እወ<br>2. ኣይፋሉን	መልሲ<br>2→507	
506	ንሕቶ ቁ 505 መልሱ እወ እንተኮይኑ፣ እቲ መዝገብ	1. እወ<br>2. ኣይፋሉን		

	ትጥቀማሉ ዶ?			
507	ወርሓዊ ናይ ሕማም ዓባይ ሰዕል ሪፖርት መግበሪ ቅጥዒ ኣለክን ዶ?(**ብምርኣይ ይረጋገፅ**)	1. እወ<br>2. ኣይፋሉን		
506	እዞም ዝስዕቡ ኢ.ደመ.ጋብርን ንትምህርታዊ ሓበሬታ/ ርክብን ባህሪያዊ ለውጢ ዘገልገሉ መሳርሕታትን ማንዋልን ኣለዉኽን ዶ?(**ምህላዎም ብምርኣይ ይረጋገፅ ዘለዉ ኩሎምይከበበሎም**)	1. ናይ ቲቢ.ናይ ጁባ ኢ.ደ-መ.ጋብር(ብቋንቋ ትግርኛ)<br>2. ሕማም ዓባይ ሰዓልመፈተሺ ኢ.ደመ.ጋብር<br>3. ዝተዋደደተሃድሶ ስልጠና ናይ ቲቢ.ን ስጋ ድዌትን ማንዋል<br>4. ሕ/ሰብ ክንክን ቲቢ ጋይድላይን<br>5. ካርዲ ናይ ሓጋዛይ ሕክምና ቲቢ<br>6. ናይ ቲቢ ፖስተር<br>7. ናይቲቢ ቡልቸር/ቡክሌት<br>8. ናይቲቢ ፊልብቻርት<br>9. ናይቲቢ ስራሕቲ ዝሓዘ ድምፅን ቪድዮን መምሃሪ ሓገዝ		

ብጣዕሚ የቐንየለይ!!!
