# Supplementary material for: Health extension workers contribution on tuberculosis case notification in Tigray region, Northern Ethiopia: A concurrent mixed method study
Source: PLoS One. 2022 Aug 16;17(8):e0271968. doi: 10.1371/journal.pone.0271968 (PMC9380935; doi:10.1371/journal.pone.0271968)
Supplement: S2 File — (PDF) [file pone.0271968.s003.pdf]

Focus Group Discussion interview guide

Part I: General Information

Date of interview ___________________________________

FGD Session Code___________ Digital recording serial number____________

Interview start time: ________________Interview end time: _________________

Name of district______________________________

Part II: Points discussion with Women Development team leaders

1. **As WDA leaders do you participate in identifying and referring presumptive TB in your Kebelle**?(Probe: house to house visit, discussion on TB sign and symptoms in WDA meeting, How HEWS communicate the about TB and how they communicate to households)
2. **How do you identify and refer presumptive TB cases in your community**? (Probe: how do you link the presumptive TB case to HEWs, what are the **challenges** in presumptive TB case identification and referral in your kebele)
3. **What can be done differently to improve the presumptive TB case identification and referral in your kebele?**

Thank you for your time!
