# Supplementary material for: Health extension workers contribution on tuberculosis case notification in Tigray region, Northern Ethiopia: A concurrent mixed method study
Source: PLoS One. 2022 Aug 16;17(8):e0271968. doi: 10.1371/journal.pone.0271968 (PMC9380935; doi:10.1371/journal.pone.0271968)
Supplement: S3 File — (PDF) [file pone.0271968.s004.pdf]

Key informant interview guide

Part I: General Information

a. Date of interview ____________________________________

b. KII Session Code___________ Digital recording serial number____________

c. Interview start time: ________________Interview end time: _________________

d. Name of the organization ____________________

e. Position ________________

f. Work experience ____________

II. Interview details /points

1. **What do you think are the role of HEWs in TB Program**? Probe for their engagement in community mobilization on TB, Presumptive TB identification and referral, TB contact screening, TB treatment follow up, LTFU tracing, etc.
2. **How do you communicate with HEWs about TB related issues? Identification, referral linkages, availability of drugs, etc**
3. **How do HEWs identify presumptive TB cases in the community**/ in your PHCU?
4. How do HEWs refer presumptive TB cases in your PHCU/ Woreda?
5. **What do you think are the operational challenges in Presumptive TB case identification and referral in your PHCU/ Woreda?**
6. **What can be done differently to improve the Presumptive TB case identification and referral in your PHCU/ Woreda? What do you think should be the role of different actors(WDGL,HEW, Kebele administration, CBO, PHCU, Woreda Health Office)**

Thank you for your time!
